# Porphyrins and Metalloporphyrins: Potential Hypoxic Agents

**DOI:** 10.1155/MBD.1996.85

**Published:** 1996

**Authors:** B. R. James, G. G. Meng, J. J. Posakony, J. A. Ravensbergen, C. J. Ware, K. A. Skov

**Affiliations:** 1 Dept. of Chemistry University of British Columbia Vancouver V6T 1Z1 Canada; 2 BC Cancer Research Centre Vancouver V5Z 1L3 Canada

## Abstract

Synthetic water-soluble porphyrins and their metalloporphyrin derivatives with Co(III), Cu(II), Ru(II) and Pt(II), containing various functional groups within the *meso*-positions of the porphyrin, were synthesised and evaluated as hypoxic agents, especially as cytotoxins and radiosensitisers. Cobalt complexes of the porphyrins containing positively charged methylpyridinium groups showed selective toxicity toward hypoxic Chinese Hamster Ovary (CHO) cells. The Co(III) complexes of the cationic and the anionic porphyrins are all weak radiosensitisers toward hypoxic cells, the highest sensitisation enhancement ratio (SER = 1.22, at 50 μM) being with a porphyrin complex containing a *cis*-arrangement of two nitro and two methylpyridinium *meso*-substituents. A copper complex of a tetracationic porphyrin showed slight radiosensitisation activity with an SER value of about 1.1. The other metalloporphyrins showed no hypoxic selectivity or radiosensitisation activity. In total, over 50 porphyrin free bases have been synthesised, of which half are water-soluble and have been metallated; thus, the chemistry is now in place for further development of water-soluble hypoxic agents.


### PORPHYRINS AND METALLOPORPHYRINS: POTENTIAL HYPOXIC AGENTS

B.R. James,[1,*] G.G. Meng,[1,2] J.J. Posakony,[1,2]

J.A. Ravensbergen,[1,2] C.J. Ware,[1,2] and K.A. Skov[2,*]

[1] Dept. of Chemistry, University of British Columbia, Vancouver, Canada V6T 1Z1
[2] BC Cancer Research Centre, Vancouver, Canada V5Z 1L3

**Abstract**
Synthetic water-soluble porphyrins and their metalloporphyrin derivatives with Co(III), Cu(II), Ru(II) and Pt(II), containing various functional groups within the *meso*-positions of the porphyrin, were synthesised and evaluated as hypoxic agents, especially as cytotoxins and radiosensitisers. Cobalt complexes of the porphyrins containing positively charged methylpyridinium groups showed selective toxicity toward hypoxic Chinese Hamster Ovary (CHO) cells. The Co(III) complexes of the cationic and the anionic porphyrins are all weak radiosensitisers toward hypoxic cells, the highest sensitisation enhancement ratio (SER = 1.22, at 50 μM) being with a porphyrin complex containing a *cis*-arrangement of two nitro and two methylpyridinium *meso*-substituents. A copper complex of a tetracationic porphyrin showed slight radiosensitisation activity with an SER value of about 1.1. The other metalloporphyrins showed no hypoxic selectivity or radiosensitisation activity. In total, over 50 porphyrin free bases have been synthesised, of which half are water-soluble and have been metallated; thus, the chemistry is now in place for further development of water-soluble hypoxic agents.

**Introduction**
Synthetic, water-soluble porphyrins have been reported to accumulate in tumour tissue [1] and such compounds containing methylpyridinium, sulfonato or carboxylato substituents, and their metal complexes, have been reported to be effective radiosensitisers [2]. The potential use of synthetic porphyrins as hypoxia selective radiosensitisers may overcome a limitation of radiotherapy which is the lack of selectivity toward tumour cells, especially toward hypoxic tumour cells. Appropriate porphyrins also have the potential for other aspects of cancer treatments, including photodynamic therapy [3], chemotherapy [4,5], boron neutron capture [6], and magnetic resonance imaging [7].

We have initiated a program to design porphyrins [8,9] containing functional groups within *meso* -phenyl or pyridyl substituents (positions 5,10,15,and 20, see Figure 1). It has been reported that nitro groups play a key role for the hypoxic toxicity and radiosensitising activities of nitro-aromatic compounds such as nitroimidazoles, due to their electron affinities [10]. We have introduced the nitro ($NO_2$) group, the nitroso group (-NO) into the porphyrin structures in order to increase the electron affinities, and the positively charged methylpyridinium group to improve solubility properties of the resultant compounds with the expectation that this may lead to better hypoxia selective and radiosensitising agents. Finally, considerable interest in nitroaromatics for detection and quantitation of hypoxia [11] leads us also to explore the use of porphyrins with an antigenic "tag" similar to that developed by Lord et al. [11], as it has been shown that the antibodies they have developed can recognize related compounds [12].

Other substituents are also under consideration for improved behaviours of porphyrins with regard to antitumour activities; for example, $-NH_2$ and pyridyl substituents might be beneficial for the interaction with DNA because these groups have the potential for hydrogen-bond formation with the DNA bases. The *meso*-phenyl and -pyridyl substituents can also be further functionalized to give water-solubility by sulfonation or N-methylation, respectively [8]. The positively charged methylpyridinium group may also facilitate interaction with the negatively charged DNA molecules and cell membranes. The structures of some of the porphyrins synthesised in this program are shown in Figure 1.

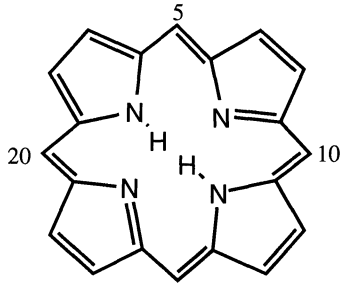

1. Tet(MPy)P = 5,10,15,20-tetrakis(4-methylpyridinium)porphyrin
2. T(MPy)PhP = 5,10,15-tris(4-methylpyridinium)-20-(4-phenyl)porphyrin
3. T(MPy)(NPh)P = 5,10,15-tris(4-methylpyridinium)-20-(4-nitrophenyl)porphyrin
4. *cis*-B(MPy)B(NPh)P = 5,10-bis(4-methylpyridinium)-15,20-bis(4-nitrophenyl)porphyrin
5. *trans*-B(MPy)B(NPh)P = 5,15-bis(4-methylpyridinium)-10,20-bis(4-nitrophenyl)porphyrin
6. Tet(SPh)P = 5,10,15,20-tetrakis(4-sulfonatophenyl)porphyrin
7. PyT(SPh)P = 5-(4-pyridyl)-10,15,20-tris(4-sulfonatophenyl)porphyrin
8. (APh)T(SPh)P = 5-(4-aminophenyl)-10,15,20-tris(4-sulfonatophenyl)porphyrin
9. *cis*-(NPh)PyB(SPh)P=5-(4-nitrophenyl)-10-(4-pyridyl)-15,20-bis(4-sulfonatophenyl)porphyrin

**Figure 1.** Examples of water-soluble porphyrin free-bases. *[APh = 4-aminophenyl, MPy = 4-methylpyridinium, NPh = 4-nitrophenyl, P = porphyrin, Py = 4-pyridyl, SPh = 4-sulfonatophenyl; B=bis, T = tris, Tet = tetrakis].* Formation of a metalloporphyrin requires loss of the two pyrrole-N protons; formulations such as Pt-**1** imply that **1** is now the dianion of the free-base porphyrin.

The additional advantages of metal compounds as radiosensitisers and cytotoxins have been previously summarised [13,14]. In terms of synthesising metalloporphyrins, we have included Pt and Ru derivatives because complexes of these metals are the most widely recognized for antitumour activity with mechanisms that require binding of the metal to DNA [14]. We have also included Co and Cu derivatives because complexes of these metals with other porphyrin ligands have been reported to yield better radiosensitisation properties than corresponding complexes with other metals [2]. This paper, as a continuation of our earlier reports [8,9], describes *in vitro* data for these metalloporphyrins with respect to accumulation in cells, cytotoxicity, radiosensitisation, and DNA binding properties.

### Materials and Methods

### *Synthesis and Characterisation*

The water-soluble porphyrins were synthesised via our recently reported methods [8,9]. The cationic porphyrins **4** and **5** were isolated as dichloride salts, **2** and **3** as trichlorides and **1** as a tetrachloride salt. Of the anionic porphyrins, **6** was isolated as a tetrasodium salt, **7** and **8** as trisodium salts, and **9** as a disodium salt. These salts were used for syntheses of the metalloporphyrins.

Metallations of the porphyrins were effected using as precursors *cis*-$Pt(H_2O)_2(DMSO)_2^{2+}$, $Ru(DMF)_6^{3+}$, or simple salts of the second row metals Co(II) and Cu(II). The Co(III) complexes of the cationic porphyrins **1**- **4** were isolated as chloride salts, while those of the anionic porphyrins **6-9** were isolated as sodium salts. The Cu(II) complexes of **1-3** were isolated as chloride salts; those of the anionic porphyrins **6-8** were isolated as sodium salts. The Co(III) species in aqueous solution were shown to be six-coordinate with two axial aquo ligands; the Cu(II) complexes were isolated as four-coordinate species [15]. The four-coordinate Pt(II) complexes of **1**, and **3-5** were first isolated as hexafluorophosphates, and the tetracationic and tricationic species (Pt-**1** and Pt-**3**) were then converted in aqueous solution to the chloride salts [16]. The other two hexafluorophosphates (Pt-**4** and Pt-**5**) were not soluble enough in water for the conversion, and were thus not tested in *in vitro* studies. The Ru(II) complexes of porphyrin **6** were isolated as $Na_4$[Ru(**6**)(CO)DMF] and $Na_4[Ru(\mathbf{6})(DMSO)_2]$ [17]. The metalloporphyrins were characterised by elemental analysis, and UV/vis-, mass-, IR-, and $^1$H- and $^{195}$Pt-NMR spectroscopies; the details of the inorganic chemistry will be reported elsewhere.

*Accumulation, Toxicity, Radiosensitisation and DNA Binding Assays*

Accumulations of the metalloporphyrins by Chinese hamster ovary (CHO) cells were determined by atomic absorption spectroscopy for detection of the metal content in the cells [18,19].

Toxicities toward the CHO cells in oxic or hypoxic conditions were performed by incubating a cell suspension in α-medium containing the dissolved metalloporphyrin drug; the cells then were washed and plated for development of colonies (7 days) as described elsewhere [8,19]. Each experiment was repeated three times and the average results are reported.

For radiosensitisation tests, the CHO cells were incubated for 1 h with medium containing a dissolved drug, and then cooled to 0 °C before irradiation using an X-ray source; after irradiation, the cells were washed free of drug and plated for development of colonies as described previously [8,19]. Plating efficiency was calculated by dividing the number of colonies by the number of cells plated (again in triplicate).

Metalloporphyrins were tested for binding to DNA by means of a plasmid binding assay [20]; DNA adducts were also tested for recognition by HMG (high mobile group) proteins [21].

### Results and Discussion

*Accumulation*

Significant amounts of Pt accumulated in the CHO cells after the cells were incubated with the Pt-**1** or Pt-**3** complexes at 100 μM under air or nitrogen; after 6 h incubation, both complexes showed about 50% favoured accumulation in hypoxic cells, with the Pt contents reaching 230 and 480 ng/($10^6$ cell) under nitrogen for Pt-**1** and Pt-**3**, respectively. The favoured accumulation of Pt-**3** over Pt-**1** presumably results from its lower charge (+3 vs. +4), perhaps because such a species more readily traverses the hydrophobic interior of the cell membrane. A similar but more pronounced effect of the charge on cell accumulation has been reported previously for the free-base porphyrins **1** and **3** [8]. The anionic Ru(**6**)(CO)(DMF) complex accumulates in CHO cells but to a level an order of magnitude lower than the cationic Pt species [e.g., ~ 6 ng/($10^6$ cell) under corresponding conditions]; nevertheless, the value is comparable to that found for cisplatin [19]. Decreased accumulation for anionic species (vs. cationic ones) is consistent with the fact that cell membranes are negatively charged.

*Toxicity*

The toxicities of the aqueous buffer-soluble Co(III) and Cu(II) complexes of **1**-**3** and **6**-**8**, the Ru(II) complexes of **6**, and the Pt(II) complexes of **1** and **3**, were all tested with CHO cells at 100 μM for 1-3 h incubation at 37 °C under hypoxic or oxic conditions. The three cationic Co(III) complexes of **1**-**3** showed similar selective toxicities to the hypoxic cells, while being essentially non-toxic toward oxic cells; the survival of the hypoxic cells incubated with these compounds for 3 h was about 40% (compared with 85-90% for controls, and aerobic treament). A representative graph of the survival curves is shown in Figure 2. Other metalloporphyrins were non-toxic under oxic or hypoxic conditions. The cationic Co(III) porphyrins almost certainly have higher reduction potentials than the other tested metalloporphyrins, and the data would then be consistent with the correlation between reduction potentials and toxicity established for a series of nitroimidazoles [10]. The mechanism for the hypoxic selectivity of cobalt(III) complexes of aliphatic mustards is considered to involve reduction of the substitution-inert Co(III) complexes to the kinetically labile Co(II) complexes, and then release of the toxic mustard ligands [22]. A different mechanism must be involved for the cationic Co(III) porphyrin complexes, because Co(II) metalloporphyrins are not readily de-metallated and, in any case, the porphyrin ligands are not toxic [8]. Unfortunately, the selective toxicities are insufficient for potential clinical use. Disappointingly, no increased selectivity was seen for the $NO_2$-containing complexes (or within the free-base porphyrins [8]), while nitroimidazoles are known to show much higher selectivities than imidazole. A Co(III) complex of **1** at 100 μM and 1 h incubation has been reported to be slightly toxic (50% survival) in Chinese hamster fibroblast (V79N) cells under oxic conditions [2]; at these conditions, we found essentially no toxicity of this complex toward CHO cells.

*Radiosensitisation*

The Co(III) complexes of **1**-**3** and **6**-**8**, the Cu(II) complex of **1**, the Pt(II) complexes of **1** and **3** and the Ru(II)(**6**)$(DMSO)_2$ complex were tested as radiosensitisers at 100 μM under hypoxic conditions in α-medium with or without serum. The less soluble Co(III) complexes of porphyrins **4** and **9** were tested at 50 μM concentration. The values of the sensitisation enhancement ratio, SER (radiation dose without drug/radiation dose with drug), were calculated at 1% survival. In contrast to the porphyrin free-bases which showed SER values of 0.92-1.04 [8], the Co(III) and Cu(II) complexes show weak radiosensitiser behaviour with SER values of 1.08-1.22 in a medium containing no serum, and 1.05-1.15 in a serum-containing medium. The cationic Co(III) complexes showed higher SER values than the anionic Co(III) complexes, and the highest value (1.22) was obtained from the Co(III)-**4** species, which contains two methylpyridinium and two nitrophenyl groups (see Figure 2).

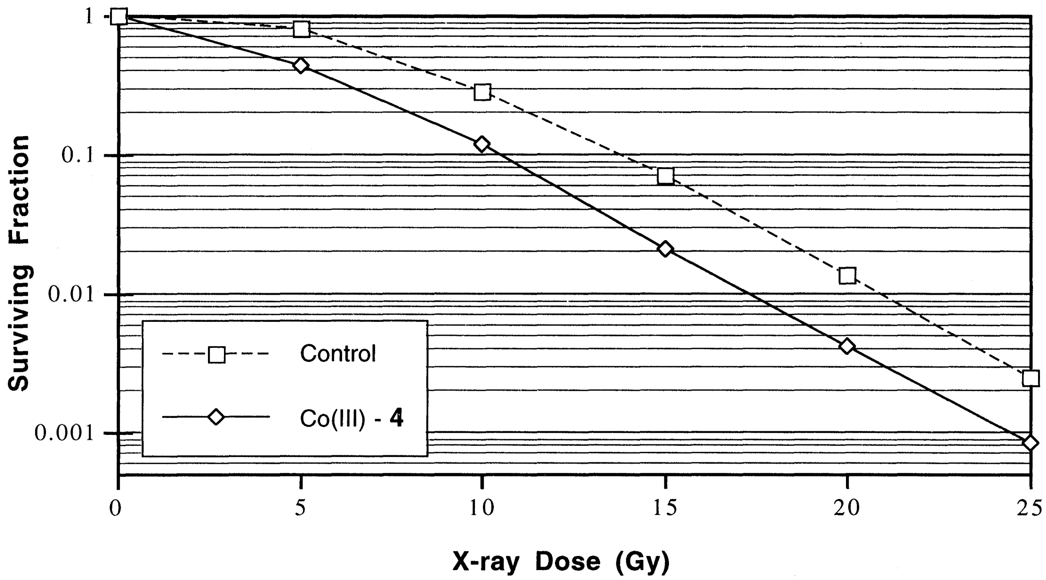

**Figure 2.** Surviving Fraction curves for radiosensitisation by Co-**4**; hypoxic conditions in α medium without serum, SER = 1.22 at 1% survival.

Although the SER value is not of interest for clinical use, the findings suggest that the introduction of nitro and/or positively charged substituents should increase radiosensitisation activities of metalloporphyrins, at least for Co(III) species. The Cu(II) complex showed SER values of 1.08 and 1.14 in a medium with or without serum, respectively. The Pt(II) and Ru(II) complexes, like the porphyrin free-bases [8], show no radiosensitisation activity (SER = 0.90-1.0). Serum has been reported to reduce cellular accumulation of Photofrin II (hematoporphyrin derivatives) [23], and thus may be a factor contributing to the somewhat lower SER values obtained in the serum-containing medium. Cobalt (III) complexes of **1** and **6**, and a copper (II) complex of **1**, were reported to be effective radiosensitisers for Chinese hamster fibroblast (V79N) cells, with SER values up to 2.4 [2]. The differences from our findings may result from differences in experimental conditions, cell lines, monolayers versus suspension, and medium (salt vs α). The characterisation, including purity level, of the complexes was not reported in the earlier work, and differences in the nature of the complexes used could also be important. In both studies, the free-base porphyrins **1** and **6** are ineffective as radiosensitisers [2,8].

*DNA Binding*

Both Ru(II) complexes of **6** do bind to DNA (at BamH1, but not EcoR1, binding sites) but are much less effective inhibitors than cisplatin for DNA damage repair; the interaction of the Ru complexes with DNA was not recognised by an HMG protein (unlike cisplatin [21,24], and *cis-* and *trans-* $RuCl_2(DMSO)_4$ [25]. The detailed mechanisms for these interactions remain to be substantiated.

In conclusion, of a range of water-soluble metalloporphyrin species, Co(III) complexes of some cationic methylpyridinium-substituted porphyrins (sometimes containing also nitrophenyl substituents) exhibit some selective toxicity and radiosensitising activity toward CHO cells.

**Acknowledgments**

We thank the Natural Sciences and Engineering Research Council and the Medical Research Council of Canada for financial support, and H. Adomat and H. Zhou for technical assistance.

### References

1. Winkelman, J.W. In *Methods in Porphyrin Photosensitization*, Kessel, D. (Ed), Plenum Press, New York, 1990, p. 91.
2. O'Hara, J.A.; Douple, E.B.; Abrams, M.J.; Picker, D.J.; Giandomenico, C.M.; Vollano, J.F. *Int. J. Radiat. Oncol. Biol. Phys.*, 1989, **16**, 1049.
3. Pondey, R.K.; Majchrzycki, D.F.; Smith, K.M.; Dougherty, T.J. in *Photodynamic Therapy: Mechanisms*, Dougherty, T.J. (Ed), SPIE: Bellingham, 1989, p. 164.
4. Sun, Y.; Martell, A.E.; Chen, D.; Macgarlane, R.D.; McNeal, C.J. *J. Heterocyclic Chem.*, 1989, **23**, 1565.
5. Ding, L.; Etemad-Moghadam, G.; Cros, S.; Meunier, B. *Biochemistry*, 1990, **29**, 7868.
6. Hill, J.S.; Kahl, S.B.; Kaye, A.H.; Stylli, S.S.; Koo, M.; Gonzales, M.F.; Vardaxis, N.J.; Johnson, C.I. *Proc. Natl. Acad. Sci. USA*, 1992, **89**, 1785.
7. Place, D.A.; Faustino, P.J.; Berghmans, K.K.; van Zijl, P.C.M.; Chesnick, A.S.; Cohen, J.S.; *Invest. Radiol.*, 1990, **25**, S69.
8. Meng, G.G.; James, B.R.; Skov, K.A.; Korbelik, M. *Can. J. Chem.*, 1994, **72**, 2447.
9. Meng, G.G.; James, B.R.; Skov, K.A. *Can. J. Chem.* , 1994, **72**, 1894.
10. Jenkins, T.C. In *The Chemistry of Antitumour Agents*, Wilman, D.E.V. (Ed), Blackie, Glasgow, 1990, p. 342.
11. Lord, E.M.; Harwell, L.; Koch, C. *Cancer Research*, 1993, **53**, 5721.
12. Matthews, J.B.; Adomat, H.; Farrell, N.; King, P.; Koch, C.; Lord, E.; Palcic, B.; Poulin, N.; Sangulin, J.; Skov, K. *British Journal of Cancer.*, 1996, in press.
13. Skov, K.A. *Radiat. Res.*, 1987, **112**, 217.
14. Farrell, N.P. In *Transition Metal Complexes as Drugs and Chemotherapeutic Agents*, Kluwer Academic Publishers, Dordrecht, 1989, Chapters 1 & 6.
15. Meng, G.G. *Ph.D. Dissertation*, University of British Columbia, 1993.
16. Ravensbergen, J.A. *M. Sc. Dissertation*, University of British Columbia, 1993.
17. Ware, C.J. *M.Sc. Dissertation*, University of British Columbia, 1994.
18. Korbelik, M.; Krosl, G.; Adomat, H.; Skov, K.A. *Photochem. and Photobiol.*, 1993, **58**, 670.
19. Chan, P.K.L.; Skov K.A.; Farrell, N.P.; James, B.R. *Int. J. Radiat. Oncol. Biol. Phys.*, 1986, **12**, 1059.
20. Skov, K.A.; Adomat, H.; Konway, D.C.; Farrell, N.P. *Chemico-Biol. Inter.*, 1987, **62**, 117.
21. Marples, B.; Adomat, H.; Billings, P.C.; Farrell, N.P.; Koch, C.J.; Skov, K.A. *Anti-Cancer Drug Design*, 1994, **9**, 389.
22. Ware, D.C.; Palmer, B.D.; Wilson, W.R.; Denny, W.A. *J. Med. Chem.*, 1993, **36**, 1839.
23. Korbelik, M. *Photochem. Photobiol.*, 1992, **56**, 391.
24. Billings, P.C.; Davis, R.J.; Engelsberg, B.N.; Skov, K.A.; Hughes, E.N. *Biochem. Biophys. Res. Commun.*, 1992, **188**, 1286.
25. Yapp, D.T.T. *Ph.D. Dissertation,* University of British Columbia, 1993.

**Received: February 13, 1996 - Accepted: February 24, 1996 - Received in revised camera-ready format: March 13, 1996**

